# Utility of Magnetocardiography and Stress Speckle Tracking in Detection of Coronary Artery Disease

**DOI:** 10.3390/diagnostics14171893

**Published:** 2024-08-28

**Authors:** Ahmed Saleh, Johannes Brachmann

**Affiliations:** 1 Cardiology Department, Centre Hospitalier du Nord, L-9080 Ettelbruck, Luxembourg; 2 RegioMed Kliniken GmbH, D-96450 Coburg, Germany

**Keywords:** coronary artery disease, ischemia, speckle tacking, magnetocardiography, strain rate, stress echocardiography

## Abstract

**Introduction:** Coronary artery disease (CAD) is a leading cause of death and disability in developed countries. While exercise testing is recommended for diagnosing stable angina pectoris, its limited sensitivity and specificity have been questioned. Myocardial strain is a promising predictor of significant CAD. **Aim:** To evaluate the utility of myocardial strain obtained by 2D speckle tracking at rest and under stress combined with stress magnetocardiography for detecting CAD in patients with stable or low-risk unstable angina pectoris. **Methods:** A total of 108 patients meeting the inclusion criteria underwent coronary angiography within 48 h of admission. Myocardial strain was assessed using 2D speckle tracking at rest and during dobutamine stress alongside stress magnetocardiography. **Results:** Global longitudinal strain at stress showed a moderate correlation with significant CAD (r = 0.41, *p* <0.0001) and with coronary occlusion severity (r = 0.62, *p* <0.0001). Strain at stress had a sensitivity of 74.1% and specificity of 76.7% for detecting CAD at a cut-off value of −19.1. The ST fluctuation rate from magnetocardiography demonstrated the highest sensitivity for CAD detection. **Conclusions:** Longitudinal strain parameters and stress magnetocardiography are effective non-invasive methods for predicting CAD in patients with stable angina, potentially reducing the need for invasive assessments.

## 1. Introduction

Coronary artery disease is a significant contributor to mortality and impairment in industrialized nations. While the fatality rate associated with this ailment has steadily decreased in recent years in Western societies, it still accounts for approximately one-third of all fatalities among individuals over 35 years old [1,2,3]. Exercise testing is commonly advised as an initial diagnostic examination for individuals with stable angina pectoris, despite criticisms of its restricted sensitivity and specificity [4,5,6]. Two-dimensional speckle tracking echocardiography (2D STE) is a modern method of cardiac imaging used to evaluate cardiac motion by tracing ultrasonic speckles in grayscale 2D images from frame to frame. It is recognized as a technology that is not significantly affected by the angle of observation and has the ability to assess global and regional strain. The analysis of myocardial strain using 2D speckle tracking during rest has shown potential in predicting the presence of significant coronary artery disease “CAD” in patients with stable angina pectoris, thus enhancing the diagnostic capabilities of traditional exercise tests [7,8,9]. Magnetocardiography (MCG) is a high-resolution technique for recording the electromagnetic activity of the heart which is non-invasive, non-contact, and free from radiation [10,11,12]. MCG has been investigated as a way to identify clinically important CAD. It provides an assessment of ischemic status that aligns with the percentage change in ST-segment fluctuation score [12,13]. The objective of this research was to evaluate the effectiveness of myocardial strain measured using 2D speckle tracking during both rest and stress, along with a new analysis technique for MCG stress testing, in identifying coronary artery disease.

## 2. Methods

Patients presenting with typical chest pain were admitted to the chest pain unit (CPU) at Coburg hospital in Germany. A total of 320 patients with stable and unstable angina (TIMI risk score 0–1) underwent initial screening, of which 108 individuals met the inclusion criteria and provided written consent to participate in the study. All participants were over 18 years old and suitable for stress testing. Coronary angiography was conducted within 48 h of admission to the chest pain unit. In this analysis, significant coronary stenosis was defined as ≥70% luminal obstruction, aligning with widely accepted standards despite acknowledgement that less severe stenosis could also pose cardiovascular risks. Exclusion criteria encompassed elevated troponin levels, ST elevation or depression as well dynamic ECG changes during CPU admission, a history of coronary artery disease or acute myocardial infarction, previous coronary artery bypass grafting procedures, chronic total coronary occlusion, significant valvular heart disease, end-stage renal failure, and refusal to provide written consent.

Following enrollment, both ECG and MCG were recorded simultaneously while at rest and during stress according to a standardized timetable. Dobutamine stress echocardiography (DSE) was also carried out prior to the invasive assessment with coronary angiography. All MCG data were captured before performing the coronary angiography.

### 2.1. Echocardiography and Quantification of Regional Strain

DSE was conducted using a standard protocol where dobutamine was administered intravenously in increasing doses to achieve a target heart rate. Continuous monitoring of blood pressure and ECG was performed throughout the test. The procedure was terminated upon reaching certain clinical criteria, such as achieving 85% of the age-predicted maximum heart rate, or in the presence of significant symptoms or ECG changes.

Echocardiographic images were obtained by three skilled echocardiographers using the GE Vivid E9 system. Standard 2D images from various apical and parasternal views were captured at different stages: during rest, dobutamine infusion, peak stress, and recovery. The images were optimized for speckle tracking analysis, particularly at high heart rates, to ensure accurate left ventricular assessment. Wall motion was visually analyzed by at least two blinded echocardiographers.

For quantifying regional strain, images were acquired at rest using the VividE9 scanner (GE Healthcare, Chicago, IL, USA) before coronary angiography. Longitudinal strain was analyzed offline using EchoPAC software. The endocardium was manually traced, and segments with poor tracking were excluded. Speckle tracking was performed on all three apical views, and negative global systolic longitudinal strain was calculated.

### 2.2. Magnetocardiography

The MCG was performed by a trained and experienced operator in accordance with the manufacturer’s instructions for use. The MCG data were collected using a 64-channel gradiometer system within a magnetically shielded room equipped with double relaxation oscillation superconducting quantum interference device sensors [14]. The MCG system utilizes double relaxation oscillation superconducting quantum interference device (DROSQUID) sensors [15,16]. The average noise spectral density of the entire system in the MSR room is 10 fT/√Hz at 1 Hz and 5 fT/√Hz over 100 Hz. Tangential components of the cardiomagnetic fields were measured, enabling comprehensive heart information retrieval with a relatively small sensor array area [17]. To facilitate analysis using magnetic field map variables, tri-polar field map patterns were transformed into ordinary dipolar field maps through minimum norm estimation [18]. Signal processing software allowed for automatic digital filtering, averaging, synthetic gradiometer formation, and baseline correction of acquired recordings. The MCG signals were recorded under resting conditions for 100 s at a sampling rate of 500 Hz with the patient lying supine and the SQUID’s positioned closely but not in direct contact with the left chest wall. Stress recordings were obtained during bicycle exercise testing while an independent investigator evaluated and analyzed ECG and MCG quality.

### 2.3. ST-Segment Fluctuation Score

The ST-segment fluctuation score was calculated by analyzing the characteristics of high-frequency components in magnetic signals from the heart during the plateau phase of the action potential, similarly to how it was carried out for the QRS fragmentation score [19]. This involved averaging and applying broadband filtering with a binomial bandpass filter (37 Hz–90 Hz). The fluctuation of the ST segment between the end of QRS and the beginning of T wave is quantified by summing the absolute values of differences in neighboring extrema (spans), with additional inclusion of absolute values of the first and last remaining extrema. The ST-segment fluctuation score is then obtained as this determined sum multiplied by number extrema, providing a comprehensive representation encompassing peaks and their heights within the bandpass-filtered signal-averaged magnetocardiography’s ST segment.

## 3. Statistical Analysis

All statistical analyses were conducted utilizing SPSS version 21.0 (SPSS, Inc., Chicago, IL, USA). Descriptive statistical techniques were employed to summarize the data. The results are expressed as mean ± standard deviation (SD) for continuous variables, while categorical variables are represented as counts and percentages. McNemar’s test was utilized to compare the sensitivities and specificities of exercise MCG and exercise ECG. To compare differences between two groups, either Student’s *t*-test or Mann–Whitney U-test (for non-Gaussian distributions) was applied for continuous variables, and χ_2_-test or Fisher’s exact test was used for categorical variables. For comparisons involving multiple groups, Analysis of Variance (ANOVA) was conducted, followed by a multiple comparison test for continuous variables and χ^2^-test for categorical variables. Receiver operating characteristic (ROC) curve analysis was performed to evaluate the diagnostic accuracy and establish optimal cut-off values for MCG parameters. The areas under the ROC curves (AUCs) were compared using the DeLong method. All applicable tests were two-tailed, with a significance level set at *p* < 0.05.

## 4. Results

The study included a total of 108 patients diagnosed with stable angina pectoris, all of whom were being evaluated for potential coronary angiography. The diagnosis was established through clinical evaluation, electrocardiogram (ECG) results, and laboratory tests. To measure global and regional strain rate values, dobutamine stress echocardiography and stress magnetocardiography were employed. The cohort consisted of 50% women, totaling 54 individuals. Among the participants, 87 were diagnosed with hypertension, and 52 had a prior diagnosis of diabetes before their admission. Additionally, 38% of the group, equating to 41 patients, were smokers, while 40 individuals either presented with elevated lipid profiles or were on lipid-lowering therapy. The average age of the participants was 64 years, with a standard deviation of ±10 years, and the mean body mass index was recorded at 25.4 kg/m^2^ ± 3.8. Upon admission, the average troponin levels were found to be 0.081 ng/mL ± 0.03 (Table 1 and Table 2).

Global longitudinal strain at rest obtained by speckle tracking showed a moderate correlation with the presence of significant CAD, r-value 0.59, *p*-value < 0.05. Global strain at stress revealed likewise a moderate correlation with the presence of significant CAD, r-value 0.66, *p*-value < 0.0001.

**Figure 1:**
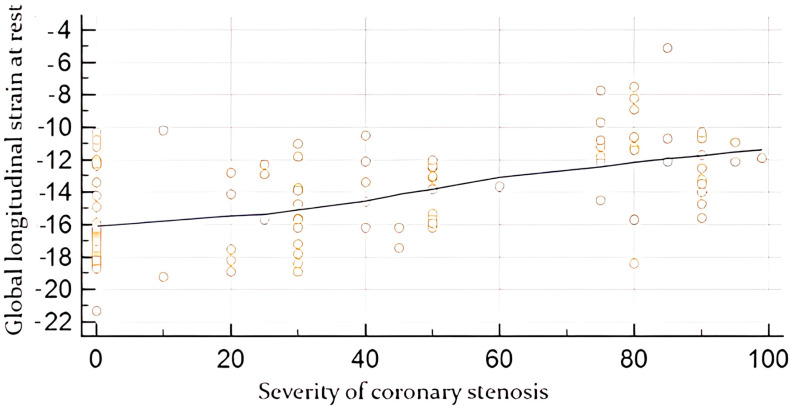
Correlation between global longitudinal strain at rest obtained by speckle tracking (in %) and the angiographic severity of coronary lesion (in %).

**Figure 2:**
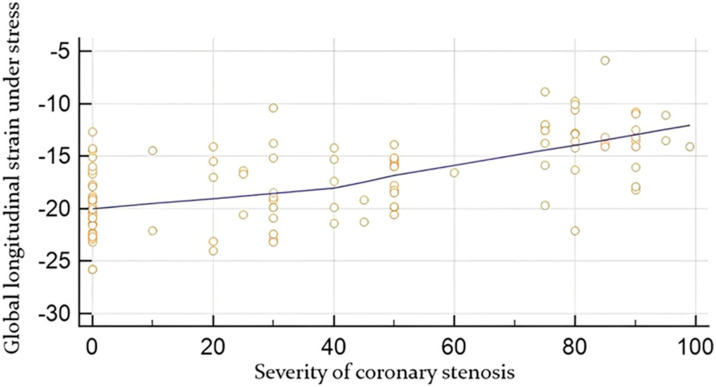
Correlation between global longitudinal strain under stress obtained by speckle tracking (in %) and the angiographic severity of coronary lesion (in %).

Moreover, a moderate correlation was found between global strain at rest and the angiographic severity of coronary lesion (in %), r-value 0.61, *p*-value < 0.0001 (Figure 1), while a moderate correlation was established between global strain under stress with the severity of coronary occlusion, r-value 0.68, *p*-value < 0.0001 (Figure 2).

In patients with one-vessel coronary artery disease (CAD), a mean global strain of −11.2 ± 2.3 was observed at rest, while a mean global strain of −13.3 ± 2.9 was recorded under stress conditions. The mean regional strain at rest was −7.9 ± 3.1, and, under stress, it was −10.9 ± 4.8. For those exhibiting angiographic signs of two-vessel CAD, the mean global strain at rest was −8.9 ± 1.5, with a mean global strain of −10.7 ± 2.4 under stress. The mean regional strain for these patients was −3.5 ± 2.3 at rest and −5.7 ± 1.7 during stress.

Patients with three-vessel disease demonstrated a mean global strain of −6.3 ± 1.1 at rest and −7.1 ± 1.5 under stress, while their mean regional strain was −1.5 ± 1.0 at rest and −3.0 ± 1.4 under stress.

The global longitudinal strain under stress exhibited a sensitivity of 85.7% and a specificity of 90.4% for identifying significant CAD with a cut-off value of −14.1 (AUC, 0.881). The application of regional strain at rest yielded a sensitivity of 77.1% and a specificity of 82.1%, whereas regional strain under stress showed a sensitivity of 82.8% and a specificity of 86.3% at cut-off values of −10 and −13, respectively.

**Figure 3:**
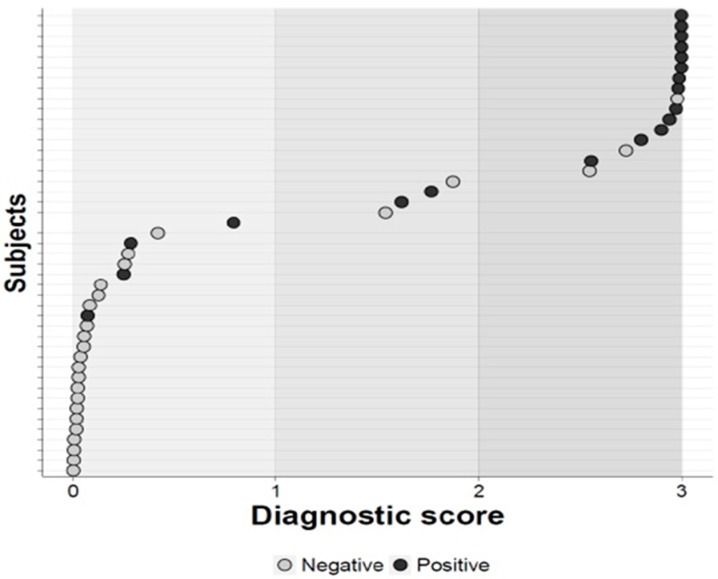
Distribution of the CAD score to predict severe CAD.

**Figure 4:**
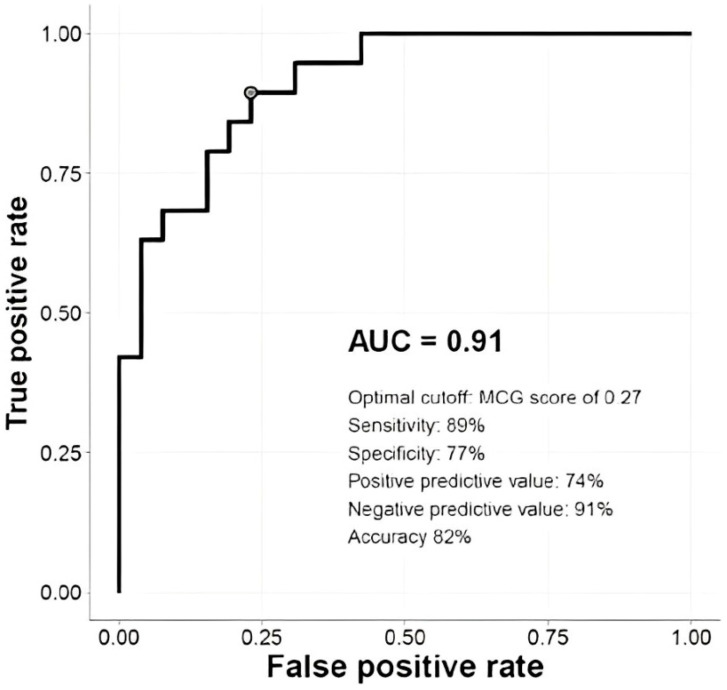
Receiver operating characteristic curve.

Additionally, the ST-segment fluctuation score derived from stress MCG demonstrated the highest sensitivity for CAD detection at 88.5% with a cut-off value of −40%, alongside a specificity of 87.6%, a positive predictive value of 77.5%, and a negative predictive value of 94.1% (Figure 3 and Figure 4).

A bimodal distribution of the magnetocardiography score was noted in the validation set with a preponderance of those with significant CAD distributed at higher scores: positive = subjects with at least one-coronary stenosis ≥ 70% (definition of significant); negative = subjects without coronary stenoses ≥ 70%.

In Figure 4, the sensitivity, specificity, positive predictive value, negative predictive value, and accuracy of MCG score are shown at the optimal cut-off.

## 5. Discussion

**Figure 5:**
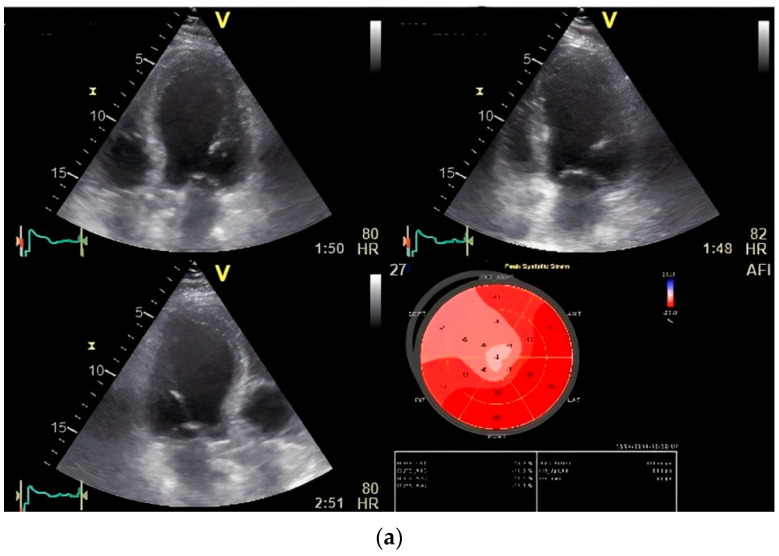

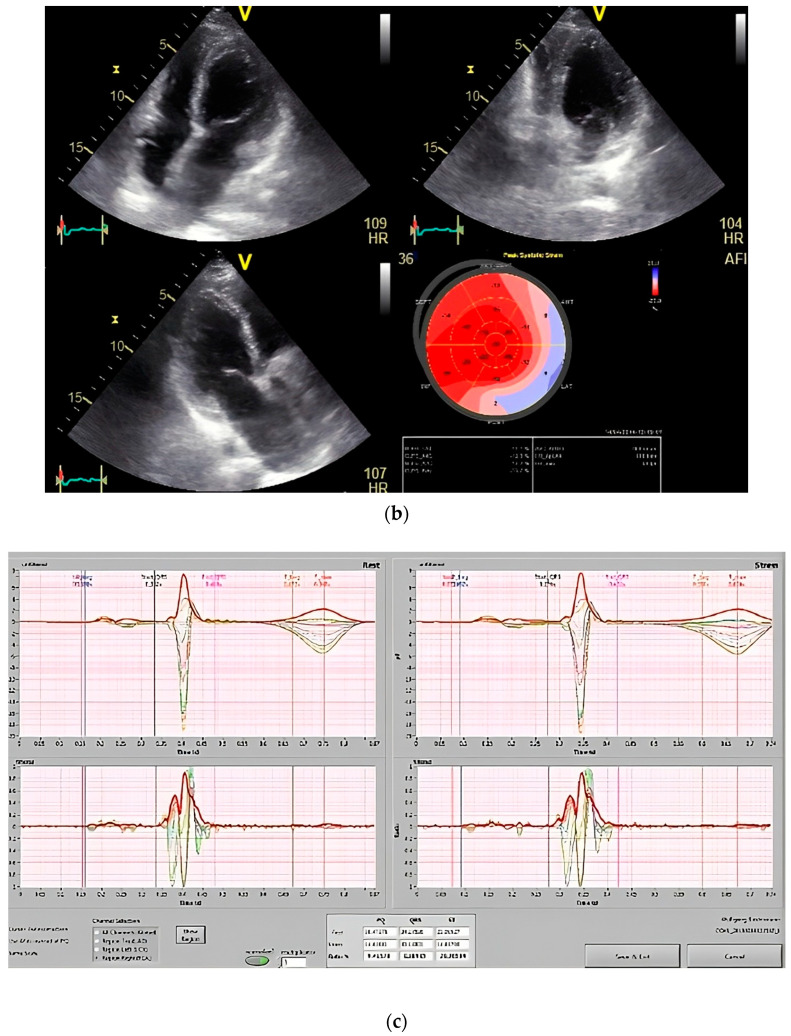
(**a**) Global longitudinal strain (GLS) by speckle tracking at rest showing decreased strain in septal region. (**b**) GLS by speckle tracking under stress (first phase stress at 20 mg/kg/min intravenous infusion of dobutamine) of the same patient showing decreased strain distributed over anterior and anterolateral region (significant left circumflex stenosis was revealed in coronary angiography). (**c**) Disturbance of T wave dispersion at stress in the same patient.

The results of our study revealed a favorable correlation between strain measurements obtained via 2D speckle tracking, both at rest and under stress, and the identification of significant coronary artery disease as confirmed by invasive coronary angiography. The strain parameters exhibited greater sensitivity and specificity during stress testing in comparison to resting states. Importantly, the ST fluctuation rate measured through magnetocardiography showed the highest level of sensitivity (Figure 5).

Furthermore, the strain parameters assessed under stress through dobutamine echocardiography were correlated with both the degree of coronary occlusion and the quantity of narrowed coronary vessels.

A study by dos Santos et al. explored the practicality of employing left ventricular longitudinal strain in an emergency department context [20]. The researchers examined 78 patients who exhibited clinical signs of unstable angina pectoris. Among the fifteen patients eligible for two-dimensional speckle tracking echocardiography (2D-STE), severe coronary lesions were confirmed via coronary cineangiography. It was also noted that patients with significant lesions in any epicardial coronary artery demonstrated a marked decrease in global strain, alongside a substantial reduction in longitudinal strain in the basal segments of the left ventricular inferior wall and lateral walls supplied by the right and circumflex coronary arteries.

These findings reinforce the existing literature, suggesting that diminished global and segmental strain are linked to the severity of myocardial ischemia, contingent upon the number of affected coronary vessels [20]. 

In a distinct study conducted in Sweden, 296 consecutive patients with suspected stable angina pectoris and normal left ventricular ejection fraction were analyzed. The results indicated that the evaluation of global longitudinal peak systolic strain at rest serves as an independent predictor of significant coronary artery disease (CAD) and significantly improves the diagnostic accuracy of an exercise test. Moreover, two-dimensional strain echocardiography demonstrated promise in identifying patients at high risk [21].

In 2018, Scharrenbroich and colleagues performed a study aimed at comparing the strain derived from speckle tracking with left ventricular ejection fraction in predicting cardiac events among patients who had experienced an acute myocardial infarction and those with established coronary artery disease. The ROC analysis for patients with coronary artery disease revealed that the addition of endocardial global circumferential strain to baseline characteristics and ejection fraction markedly enhanced the predictive capacity for cardiac events, achieving an area under the curve of 0.86 with a cut-off value of 20%, sensitivity of 79%, and specificity of 84% [22].

A cross-sectional investigation carried out in Bangladesh involved patients diagnosed with acute coronary syndrome who underwent 3D speckle tracking echocardiography prior to coronary angiography. The participants were categorized into two groups based on the presence of significant stenosis as determined by the angiographic findings [23]. 

All strain parameters exhibited a notable reduction in the cohort with significant stenosis. The analysis of the receiver operating characteristic curve demonstrated that GPSLS effectively identified patients with significant stenosis, achieving an area under the ROC curve of 0.840 (95% CI = 0.735–0.945). Utilizing a cut-off value of −13.50%, GPSLS displayed commendable sensitivity and specificity for predicting significant stenosis with sensitivity at 88.9% and specificity at 70.8%.

In a separate study by Chaikovsky et al., ischemic patients were assessed through MCG testing which included 49 healthy individuals and 51 patients with coronary artery disease (CAD). The MCG data analysis indicated that the control group had a more consistent current distribution compared to the CAD group, which presented additional current areas with altered directionality. These additional currents appeared to correlate with the anatomical regions of ischemia and were associated with the affected coronary artery. The study reported a sensitivity of 91% and a specificity of 84% for MCG [24].

A separate study presented findings from magnetocardiography (MCG) conducted on patients experiencing chest pain who underwent both stress SPECT and coronary angiography evaluations. Approximately fifty percent of the participants were assessed for chronic ischemic heart disease, while the remainder received evaluations following acute chest pain incidents. The results demonstrated a high level of diagnostic accuracy for resting MCG when compared to stress nuclear scans using obstructive coronary artery disease (CAD) as the reference standard [25]. 

In a recent investigation carried out in Germany, researchers examined the potential of magnetocardiography during resting periods to predict mortality in patients with acute chest pain. The primary outcomes of this study indicated that prolonged QTc intervals, increased serum creatinine levels, and diminished repolarization reserve identified through resting MCG were highly effective in forecasting mid-to-long-term mortality in individuals presenting with acute chest pain. The researchers concluded that the high sensitivity (90.9%) for cardiac death, specificity (85.6%), and remarkable negative predictive value (99.4%) could pave the way for advancements in clinical diagnostics [26]. 

A research team from India conducted a study comparing magnetocardiography to the treadmill test (TMT) in patients experiencing chronic chest pain and suspected coronary ischemia. The iso-field contour maps created at the peak of the T wave were analyzed between the two groups. The magnetic field angle at the T wave peak was derived from the magnetic field map (MFM) and subsequently compared across the groups, which included 12 patients with positive TMT results and 17 with negative TMT results. Abnormal contour maps, characterized by nondipole patterns or unusual orientations, were observed in 81.8% (9 out of 11) of the patients in the TMT-positive group, while only 6.8% (1 out of 17) of the patients in the TMT-negative group exhibited similar abnormalities (*p* <0.001). The researchers concluded that the presence of abnormal magnetic field angles and magnetic field maps in magnetocardiography recorded at rest can effectively indicate coronary ischemia in patients suffering from chronic chest pain, even when their resting ECG appears normal [27].

In investigations involving patients presenting with acute chest pain and suspected acute coronary syndrome (ACS), the evaluation of magnetocardiography (MCG) data—obtained either at rest or following physical exertion, in both shielded and unshielded settings—has demonstrated both qualitative and quantitative differences that aid in distinguishing between individuals with ACS and those who are healthy. Furthermore, MCG has proven effective in identifying patients who lack conclusive evidence of ACS or coronary artery disease (CAD) during diagnostic assessments. Huang and colleagues conducted a cross-sectional study to evaluate the efficacy of MCG in identifying patients who may require coronary revascularization. The performance metrics for the 12-lead electrocardiogram (ECG) included an accuracy of 67.0%, sensitivity of 62.7%, specificity of 63.5%, positive predictive value (PPV) of 70.5%, and negative predictive value (NPV) of 55.1%. In comparison, the MCG model exhibited an accuracy of 68.2%, sensitivity of 70.1%, specificity of 66.3%, PPV of 68.5%, and NPV of 67.9% [10]. 

### Limitations

1. Negative impact of heart rate variability on strain rate offline analysis led to the exclusion of numerous patients from the study, including those with atrial fibrillation due to the program’s inability to address this issue.

2. Difficulty in obtaining a clear acoustic window during echocardiography in obese patients or those with hyper-inflated thoracic walls remains a significant limitation.

3. This study was conducted at a single center and included a relatively small number of participants.

4. Patients with certain medical devices such as pacemakers, central nervous system aneurysm clips, cochlear implants, implanted neural stimulators, or insulin pumps were not suitable for examination using MCG.

5. The determination of coronary occlusion severity mainly relied on angiography criteria; therefore, only 23 patients underwent further assessment using FFR (fractional flow reserve) or IFR (instantaneous wave-free ratio).

## 6. Conclusions

Longitudinal strain measurements at rest and during stress have the potential to forecast the existence of coronary artery disease (CAD) in individuals with stable angina pectoris. Additionally, these measures could indicate the extent of coronary artery involvement before invasive diagnostic procedures, offering a non-invasive approach to better patient stratification. The sensitivity and specificity of strain parameters during stress were found to be higher than those at rest for detecting CAD, highlighting the importance of stress testing in clinical evaluations.

Moreover, the ST fluctuation score exhibited the greatest sensitivity in predicting CAD, suggesting that this parameter could serve as a valuable tool in the early identification of high-risk patients. The findings underscore the potential of these non-invasive methods not only to improve diagnostic accuracy but also to enhance decision-making in clinical practice.

Given the robustness of these results, it is strongly advised to routinely utilize both longitudinal strain measurements and ST fluctuation scores prior to invasive investigations for coronary vessels. This approach could reduce the need for unnecessary invasive procedures and ultimately improve patient outcomes. Future studies should focus on validating these findings in larger, diverse populations and explore the integration of these methods into standard diagnostic protocols.

The integration of strain measurements and ST fluctuation scores into routine clinical practice could represent a significant advancement in the early detection and management of coronary artery disease, paving the way for more personalized and effective treatment strategies.

## Figures and Tables

**Table 1:** Demographics of patients who were enrolled in the study. ACS: acute coronary syndrome. PCI: percutaneous coronary intervention.

	Number	Percentage
Gender		
Male	54	50%
Female	54	50%
Hypertension		
Non-hypertensive	21	19.50%
Hypertensive	87	80.50%
Diabetes mellitus		
Non-diabetics	56	51.90%
Diabetics	52	48.10%
Smoking		
Non-smoker	67	62%
Smoker	41	38%
Hyperlipidemia		
No	68	63%
Yes	40	37%
Previous ACS		
No	90	83.40%
Yes	18	16.60%
Previous PCI		
No	98	90.80%
Yes	10	9.20%

**Table 2:** Baseline characteristics based on presence or absence of coronary artery disease (CAD). No: number. %: percentage in each group (no-CAD or CAD group). DM: diabetes mellitus. EF: ejection fraction. GLS: global longitudinal strain.

	No CAD	CAD	*p*-Value
Age (years)	65 ± 12	63 ± 9	0.519
Men no.	41	13	0.05
Hypertension no. (%)	60(82.2)	27 (77.1)	0.353
DM no. (%)	31(42.5)	21 (60.0)	0.067
Hyperlipidemia no. (%)	28(38.4)	12 (34.3)	0.424
EF (%)	58.0 ± 6.4	48.9 ± 5.9	<0.001
Troponin (ng/mL)	0.068 ± 0.02	0.081 ± 0.07	0.315
WMA in stress echocardiography (no.)	7	31	<0.001
GLS at rest (%)	−18.4 ± 3.1	−16.9 ± 2.8	<0.001
GLS under stress (%)	−21.9 ± 3.7	−20.8 ± 3.3	0.019

## Data Availability

All data is available upon request.
